# A case of a tumor‐like condition in the optic nerve head of a pig

**DOI:** 10.1002/ccr3.1858

**Published:** 2018-10-09

**Authors:** Alexey A. Suetov, Ernest V. Boiko, Sergey I. Alekperov

**Affiliations:** ^1^ State Scientific Research Test Institute of Military Medicine St. Petersburg Russia; ^2^ St. Petersburg Branch S. Fyodorov Eye Microsurgery Federal State Institution St. Petersburg Russia; ^3^ Department of Ophthalmology Military Medical Academy St. Petersburg Russia; ^4^ Department of Ophthalmology Mechnikov North‐West State Medical University St. Petersburg Russia

**Keywords:** Bergmeister’s papilla, exudative optic neuritis, ischemic optic neuropathy, optic nerve, proliferative optic neuropathy, traumatic optic neuropathy

## Abstract

A tumor‐like condition of the optic nerve head of unknown etiology was found in a domestic pig. Clinical and histological manifestations suggest that the unusual tumor‐like condition is probably a variant of proliferative optic neuropathy caused by unknown nonspecific damage (perhaps trauma), which was received earlier.

## 1 INTRODUCTION

Among the rare changes in the optic nerve head in humans and animals, lesions that macroscopically resemble masses protruding from the optic nerve head into the vitreous body can be distinguished from tumors.^1^, ^2^ Such changes can be caused by different factors; however, in general they are called "pseudo‐tumors of the optic disk".^1^ There is little information in the literature about these lesions; therefore, their detection can be difficult to interpret and diagnose.

The purpose of this case report is to describe the clinical presentation, diagnosis, and histological features of an unusual tumor‐like condition (TLC) of the optic nerve head of a pig compared to available data and to discuss the possible causes.

## 2 CASE REPORT

**Figure 1:**
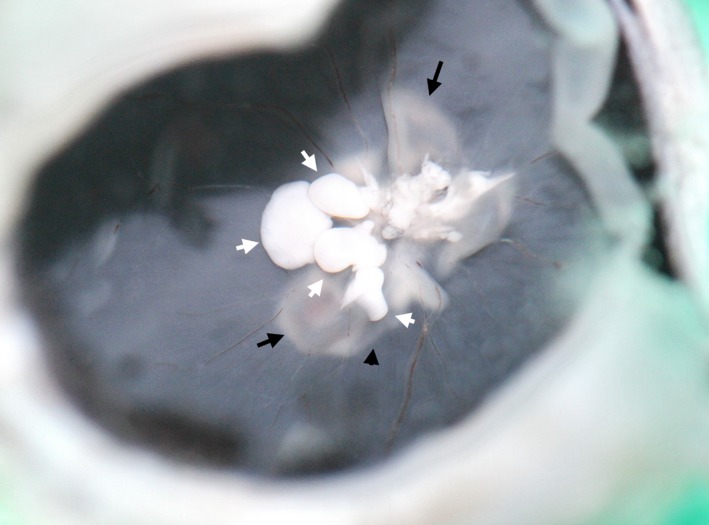
Multiple pedunculated well‐defined white nodules (white arrows) of the tumor‐like condition of the optic nerve head in a pig. In the retina around the disk, there is local folding with detached neuroepithelium (black arrows)

A TLC of the optic nerve head of the right eye (Figure 1) was found during the examination of the ocular fundus of a male domestic pig (*Sus scrofa domestica*) at the age of 10 weeks and weighing 35 kg in preparation for the experimental study of the effects of long‐term local exposition of an extremely low‐frequency magnetic field on the reproductive system. A physical examination did not reveal any significant features. There was no evidence that the animal had previously had an eye injury, head trauma, or systemic or ocular disease.

Exophthalmos was not found. A slit‐lamp examination showed no abnormality in the anterior segment and media of the right eye. For the ophthalmoscopy of the fundus, a Heine OMEGA500 binocular indirect ophthalmoscope (Heine Optotechnik, Germany) was used, and a white tumor‐like mass was visualized. This mass protruded from the optic nerve head into the vitreous cavity and had multiple pedunculated nodules; it had a clear shape and a smooth surface on the temporal side and an irregular shape and an uneven, rough surface on the superior nasal side. Around the optic disk, retinal folding and detachment of the neuroepithelium were observed, which were more pronounced at the lower edge of the optic disk. The vitreous was clear. There was no inflammatory sign in the fundus or in the vitreous. Despite the changes in branching of the retinal vessels caused by the TLC of the optic nerve head, in general, there was no sign of vascular change.

The pupil of the right eye was slightly dilated, and relative afferent pupillary defect (RAPD) was present. Results of the examination of the left eye were unremarkable.

Intraocular pressure was measured with a Reichert TonoPen‐Vet system (Reichert Inc, USA) and was 15.8 mm Hg for the right eye and 15.4 mm Hg for the left eye.

During the experiment, only the reproductive organs of the pig were exposed to magnetic fields, and no local or systemic effect was observed. The revealed TLC did not change during the experiment. The pig was euthanatized with an overdose of sodium pentobarbital (180 mg/kg) while under general anesthesia.

Following the experiment in which both eye globes were enucleated, they were placed in a 0.1 mol/L phosphate‐buffered solution containing 4% formalin at room temperature for 6 hours. Then, the eye globes were trimmed, and the eyecups with the vitreous and the optic nerve were fixed in a 0.1 mol/L phosphate‐buffered solution containing 10% formalin at 4°C for 24 hours. The fixed eye globes were dehydrated, embedded in paraffin, and cut into 4 µm thick sections. The histologic sections were stained with hematoxylin and eosin (H&E) and Picro‐Mallory trichrome. Specific cell populations were observed using immunohistochemical (IHC) staining for glial fibrillary acid protein (GFAP), CD57 (astrocytes markers), CD68 (marker of macrophages and activated microglia), and CD163 (marker of perivascular macrophages).^3^, ^4^ Manual IHC staining was performed using primary anti‐rat antibodies (Cell Marque, USA) and a polyvalent HRP‐DAB detection system (REVEAL, Spring Bioscience, USA). All sections were observed with an optical microscope.

## 3 MORPHOLOGY FEATURES

### 3.1 TLC and prelaminar portion of the optic nerve

**Figure 2:**
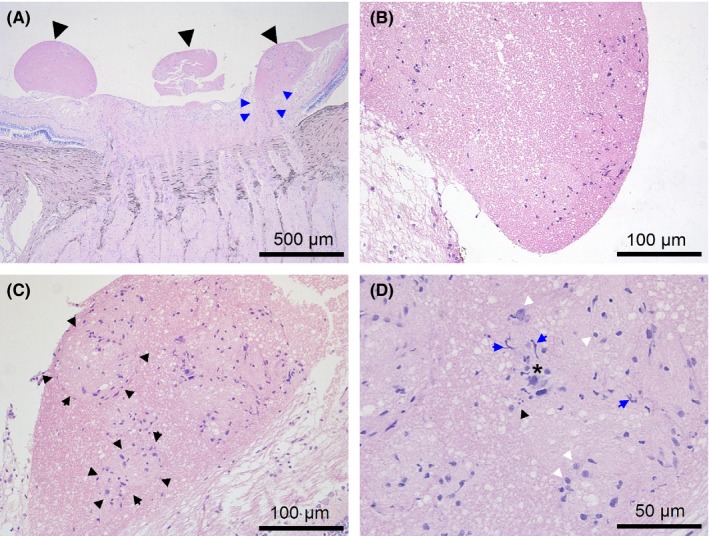
H&E stain of the tumor‐like condition of a pig. A, shows multiple pedunculated well‐defined nodules (black arrows) formed mainly from the neuroretinal rim area and borders of the optic nerve head (blue arrows). B, shows that the nodule containing a small number of cells is not encapsulated. C, shows some cell conglomerates in the nodules (the boundaries of some conglomerates are indicated by black arrows). D, shows that there are different forms of glial cells in the nodules (white and blue arrows). Cell damage in the form of pyknosis of the nuclei (black asterisk), karyolysis and cell debris (black arrows) are present in the nodules

**Figure 3:**
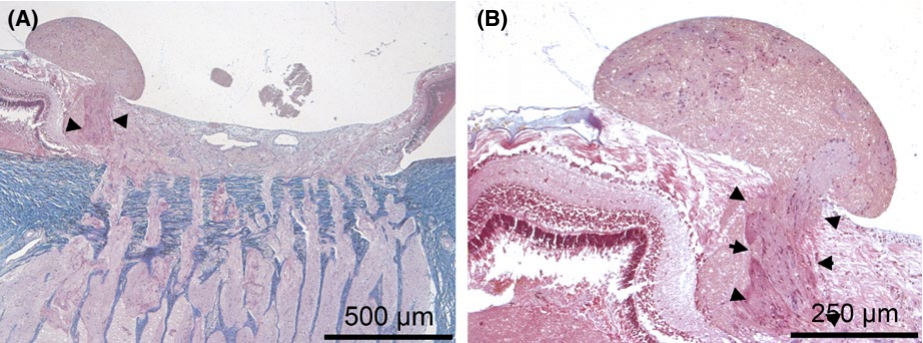
The pedunculated nodule is formed from the glial tissue of the nerve fiber layer (black arrows) and does not contain fibrous tissue, as shown by the Picro‐Mallory trichrome stain

**Figure 4:**
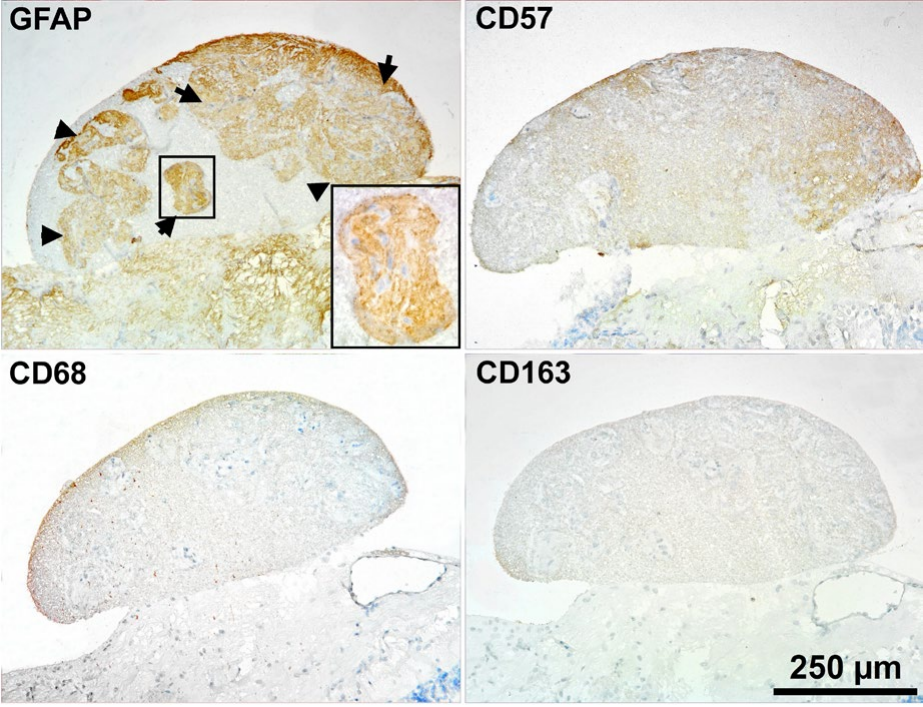
Immunohistochemical staining of the nodules of TLC shows that GFAP‐positive cells (astrocytes) are the predominant type of cells in the nodules (black arrows indicate GFAP‐positive cell conglomerates in the nodules). Staining results of glial cells in the nodules with CD57, CD68, and CD163 antibodies were negative

The TLC was similar to the hernial protrusion of the retinal nerve fiber layer and the prelaminar portion of the optic nerve. The multiple pedunculated nodules of the TLC consisted of nervous tissue with a small number of cells and differed from the surrounding nerve fiber layer by having a denser structure and more intense staining (Figures 2A,D and 3). Multiple unencapsulated nodules developed from the nerve fiber layer and were well defined (Figure 2B). From the serial sections, it was observed that the nodules were formed mainly from the neuroretinal rim area and the borders of the optic nerve head (Figure 2A). Structurally similar nodules were found in a region of the prelaminar portion and the surface nerve fiber layer of the optic nerve head; however, they did not protrude into the vitreous. Mild edema was observed in the nodules, but no sign of vascularization was detected. Individual conglomerates that were contained in the main part of all cells were detected in the nodules (Figure 2C). The staining with GFAP antibodies showed that astrocytes were the predominant type of cells in the nodules (Figure 4). The staining of glial cells in the nodules with CD57 antibodies was negative. The histology of some cells was similar to that of macrophages; however, these cells were not stained by the CD68‐antibodies or the CD163‐antibodies. A small number of CD68‐positive cells were detected in the nerve fiber layer region and prelaminar part of the optic disk (Figure 6D). In addition, signs of cell damage in the form of excessive eosinophilic cytoplasm, pyknosis of nuclei, karyolysis, and cell debris were observed (Figure 2D). In addition, microscopy images showed that neutrophils, lymphocytes, and other inflammatory cells were not in the nodules or surrounding tissues. Interestingly, negative CD68 and CD163 antibody staining of cells with signs of damage indicated both the absence of macrophages and the presence of activated microglia.

### 3.2 Lamina cribrosa and retrolaminar part of optic nerve

**Figure 5:**
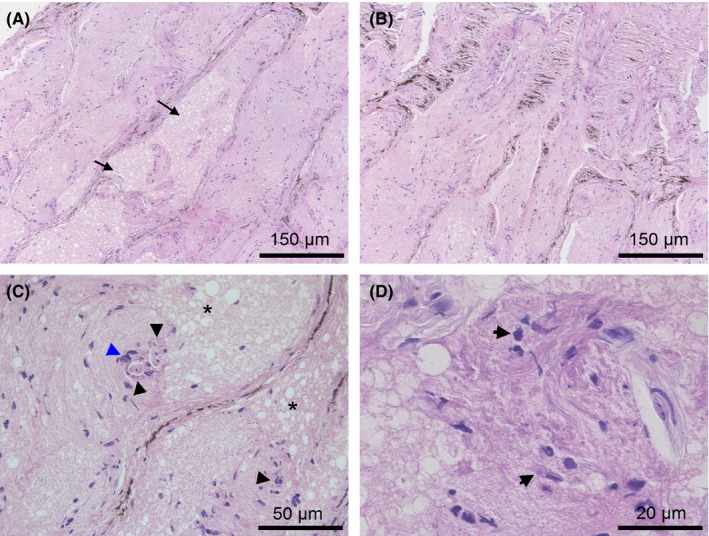
H&E staining results of the lamina cribrosa and retrolaminar part of the optic nerve. A, shows mild tissue swelling in some nerve fiber bundles (black arrows). B, shows the lamina cribrosa without any sign of damage. C, shows signs of cell injury (black arrows) and mild gliosis (blue arrows) in the retrolaminar part of the optic nerve. D, shows signs of damage of the glial cells (black arrows)

In the lamina cribrosa and retrolaminar neural tissue, bundles of nerve fibers showed signs of tissue swelling and mild or moderate gliosis and were either damaged or dead (Figure 5). No hemorrhaging or vascular abnormality was detected.

### 3.3 Juxtapapillary retina

**Figure 6:**
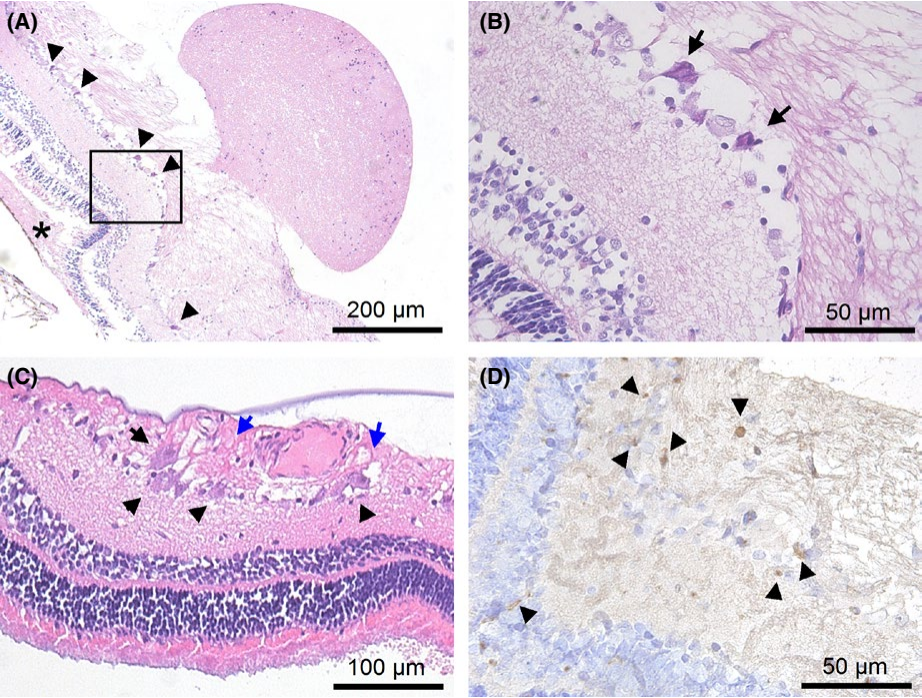
H&E (A‐C) and IHC (D) staining results of the juxtapapillary retina. A and B, show retinal cell damage (black arrows) and subretinal fluid accumulation (black asterisk) in the area where the nodules formed. C, shows retinal ganglion cells damage with an excessively shrunken eosinophilic cytoplasm (black arrows) and mild tissue swelling (blue arrows). D, shows that there are CD68‐positive cells in the retina near the nodules

In the retina and adjacent to the optic disk, there were signs of mild tissue edema, and the retinal ganglion cells were damaged with an excessively shrunken eosinophilic cytoplasm (Figure 6). Around the retinal vessels, mild perivascular edema was observed (Figure 6A,B). Under the detached neuroepithelium, there was subretinal fluid (Figure 6A). The combination of swelling and local retinal detachment caused the retina to fold visibly under the ophthalmoscope.

## 4 DIFFERENTIAL DIAGNOSIS

Developmental abnormalities, tumors, injuries, and infectious and inflammatory diseases can cause various pathological changes in the optic nerve head, resulting in the protrusion of masses into the vitreous.^1^, ^2^, ^6^, ^7^

The clinically observed TLC of the optic nerve head shares some similarities with that of persistent Bergmeister's papilla (BP), which is a congenital vascular abnormality in which the regression of neuroglial and fibrous tissues around the embryonal hyaloidal vessels of the primary vitreous is incomplete.^6^, ^9^ BP has been reported in humans and some species of mammals.^7^, ^10^ Remnants of BP can manifest as a solid, yellow‐white protuberance on the optic disk. In 90% of the cases, BP is located in the nasal optic disk sector, and physiological cup is reduced or absent.^6^ However, there were differences between the ophthalmoscopy results and the subsequent histological examination of the detected TLC. Multiple pedunculated nodules did not contain fibrous tissue or remnants of vessels; these nodules were formed mainly from the neuroretinal rim area and borders of the optic nerve head and contained astrocytes and other glial cells with some signs of damage.

Similar lesions of the optic nerve head with protruding nodules into the vitreous have been previously described in horses. In particular, proliferative optic neuropathy (PON), exudative optic neuritis (EON), ischemic optic neuropathy (ION), and traumatic optic neuropathy were observed.^2^, ^10^, ^11^

Proliferative optic neuropathy was an incidental finding in aged horses and is characterized by unilateral and non‐progressive lesions that do not affect vision or pupillary light reactions.^12^ PON manifests as a well defined, white or gray solitary mass that protrudes into the vitreous from an optic disk that otherwise appears normal.^11^, ^12^ Previously, it has been suggested that PON is a consequence of lipid storage disorder; in some cases, the nodules were described to contain conglomerates of large, thin‐walled cells with foamy cytoplasm (gitter or foam‐like cells) and nuclei that were displaced to the periphery of the cells.^8^, ^11^ These cell conglomerates partially replace normal optic nerve tissue. However, according to other hypotheses, PON may represent a reaction of glial cells to a nonspecific insult or granular cell tumor.^10^, ^13^ In addition, gitter cells were not present in the nodules in all of the described cases of PON.^11^

Exudative optic neuritis in horses is caused by severe, acute inflammation of the bilateral optic nerve.^10^ In those affected with EON, multiple oval or round yellowish bodies protrude from the borders of the optic disk and extend into the vitreous.^12^ Hemorrhaging around the optic disk and vitreous haze can be observed. The pupils are dilated, and pupillary light reactions are inhibited or absent.^10^ In some histological studies, large nodules containing gitter cells were revealed to replace most of the disk.^2^ In the surrounding retina, thick‐walled, hyalinized vessels with reduced lumen may be present. The cause of EON is unknown; presumably, EON develops in the background of various systemic diseases such as infection.^2^

In our case, although macroscopically similar, TLC was different from PON and EON. The discovered TLC was unilateral with a RAPD. There was no hemorrhaging, vascular congestion, or vitreous haze. Histologically, the lesion consisted only of nervous tissue. In the multiple protruded nodules, only a few detected cells were similar to gitter cells; however, they were negative for CD68 and CD163. There was no sign of acute inflammation or severe optic nerve atrophy. Ho)wever, the conglomerates in the nodules consisted of a few glial cells and many astrocytes, which may be due to their previous reactivation and proliferation in response to nonspecific damage.

Ischemic optic neuropathy is clinically similar to TLC and histologically similar to EON.^2^, ^10^ ION can be caused by sudden hypoxemia in the optic nerve due to trauma, thromboembolic disease, or ligation of the internal and external carotid arteries.^12^ Traumatic optic neuropathy with malacia has also been described to have a similar effect on the optic nerve head of horses.^2^, ^14^ In both conditions, initial tissue injury induced by ischemic damage led to total or segmental necrosis of the optic nerve tissue; as a result, during the acute period, tissue swelling and the presence of necrotic and gitter cells may be histologically observed.^14^ The found TLC was very similar to traumatic optic neuropathy with malacia in horses; however, in this case, there were few gitter cells or damaged cells, and there was not any vitreous haze. Perhaps these differences are due to the detection of TLC a long time after the injury.

Moreover, following acute elevation of intraocular pressure in goniodysgenesis‐related glaucoma, optic neuropathy may result in the protrusion of white nodules into the vitreous.^2^ During the acute period, the swelling of the optic nerve head is followed by protrusion into the vitreous of granular necrotic neuropil containing a few phagocytic cells. Subsequently, malacia of the optic disk neuropil with a predominance of gitter cells is observed. The optic nerve tissue becomes cavitated.

Tumors of the optic nerve such as retinal astrocytic hamartoma and medulloepithelioma of the optic nerve head are also accompanied in many cases by the formation of nodules or masses protruding into the vitreous; thus, they have been described in humans and various other mammals.^1^, ^15^, ^16^ Nevertheless, the revealed condition has no morphological features of the tumor: The nodules contain few cells, and there is no sign of proliferative activity. In addition, there is no tumor in the surrounding tissues, the growth of which can lead to disruption of axonal transport, edema, and ischemia with necrosis of the optic nerve head.

The found TLC shared no clinical or histological similarity with protrusion of the optic nerve head into the vitreous as a result of optic neuritis or papilledema.^1^, ^2^, ^7^ For example, in the optic nerve and pre‐papillary vitreous, there was no sign of inflammation, and the form of protrusion did not correspond to the prominence observed in swelling disk or optic neuritis; additionally, there was no sign of vascular congestion or hemorrhaging.

Based on the above results, we assume that the discovered TLC is most likely a variant of proliferative optic neuropathy from nonspecific damage (perhaps trauma) received earlier if hypothesis that PON can be caused by glial proliferation in response to various injuries of the optic nerve head is correct.

In conclusion, although this case involved a pig, because of the rarity of these TLCs, the results can provide a useful clinical and morphological illustration of possible changes in the optic nerve of other animals and humans.

## CONFLICT OF INTEREST

None declared.

## AUTHOR CONTRIBUTION

AA and EV: designed the concept of the clinical case report and performed the clinical examination. AA and SI: performed the pathological procedures. All authors contributed with the writing and approval of the final manuscript.
